# Contrasting Propagation of Natural Calls of Two Anuran Species from the South American Temperate Forest

**DOI:** 10.1371/journal.pone.0134498

**Published:** 2015-07-31

**Authors:** Mario Penna, Felipe N. Moreno-Gómez

**Affiliations:** Program of Physiology and Biophysics, Institute of Biomedical Sciences, Faculty of Medicine, University of Chile, Independencia 838000, Santiago, Chile; Virginia Commonwealth University, UNITED STATES

## Abstract

The acoustic adaptation hypothesis predicts that sound communication signals have an optimal relationship with animals’ native environments. However, species sharing a habitat produce signals stratified in the spectral domain and exhibit different temporal patterns resulting in acoustic niche partitioning. The diversity generated is likely to affect differently the characteristics of propagating signals. We recorded at various distances from the sound source calls of the frogs *Eupsophus calcaratus* and *E*. *emiliopugini* in the austral temperate forest where they communicate and breed syntopically. The calls of *E*. *calcaratus* have higher frequency components and lower amplitude relative to calls of *E*. *emiliopugini*, and the acoustic active space for the signals of *E*. *calcaratus* is restricted relative to *E*. *emiliopugini*. The signals of both species experience similar attenuation patterns, but calls of *E*. *calcaratus* are affected by spectral degradation to a larger extent, with linear decreases in spectral cross-correlation and in the amplitude ratio between the first two harmonics. The calls of *E*. *emiliopugini* are affected by temporal degradation as a linear decrease in amplitude modulation depth of their pulsed structure. Further studies are needed to assess the relative importance of selective and phylogenetic factors on the divergent propagation patterns reported.

## Introduction

Acoustic signals are affected by phenomena of attenuation and degradation as they propagate across natural terrestrial environments [1]. In addition, noises generated by natural biotic and abiotic sources contribute masking interference affecting the integrity of the signals [2], and these phenomena altogether alter communication efficiency.

Studies in birds and primates have reported diverse instances supporting a signal design that maximizes communication range. Such adaptations imply that signals of species from different populations have features favoring transmission in their native habitats, a prediction resulting from the acoustic adaptation hypothesis [3] [4] [5]. However, coexisting species can differ in the transmission properties of their signals as a result of constraints of diverse origin. For instance, species of relatively small sizes produce signals having higher frequencies, which are likely to suffer higher degradation and attenuation relative to lower frequency signals produced by larger animals. Although the use of high frequency signals is disadvantageous in terms of propagation, it contributes to interference avoidance. Such condition has been observed in insects, frogs and birds living in syntopy, which typically produce signals stratified in the spectral domain and exhibit different temporal patterns resulting in acoustic niche partitioning [6, 7, 8].

Studies on anuran sound communication have mostly reported a lack of relationships between signal features and vegetation coverage of their native environments of the kind reported for birds and primates [9–12], and the structure of the vocalizations is associated with the phylogenetic relationships [13–15]. Such lack of a clear optimization suggests that anuran vocalizations are designed primarily to locate mates and competitors within limited breeding areas [16].

Most studies investigating environmental adaptations of acoustic signals have compared propagation patterns of sounds among different environments in distant geographical areas (reviewed in [17–19] or among different microenvironments within the same locality (e. g. [20–22]. However, few studies have compared transmission patterns of signals with different acoustic structure from species sharing the same environment. Furthermore, surprisingly few studies of signal propagation have measured vocalizations produced by vertebrates calling in their natural habitat [23, 24]. Rather, prerecorded signals played back though loudspeakers have been used extensively [25, 26]. Such methodology yields results comparable on average to those using actual calling animals, although it underestimates the variation inherent to natural emitters [24].

In former studies we have carried out amplitude and spectral measurements of calls produced by males of *Eupsophus calcaratus* and *E*. *emiliopugini*, two species that breed in the same environment overlapping partially their reproductive periods during mid spring. Call measurements at different distances from the sound source were carried out placing a single microphone sequentially at different distances from focal subjects [27, 28]. These studies also included auditory threshold measurements, allowing us to compare the active acoustic space of both taxa, which is restricted to distances below 2 m for *E*. *calcaratus* and extends beyond 8 m for *E*. *emiliopugini*. The signals of the two *Eupsophus* species have contrasting structures: the call of *E*. *calcaratus* is a single note having a harmonic structure, susceptible to spectral degradation [27], whereas the call of *E*. *emiliopugini* has a lower frequency spectrum and a pulsed structure potentially vulnerable to temporal degradation [28]. However the procedure used for measuring calls in previous studies, does not allow recording simultaneously the same signal at different distances, and therefore does not yield precise measurements of spectral and temporal alterations affecting signals as they propagate. Simultaneous measurements of the same propagating signals need to be conducted as has been reported for Iberian toads *Alytes* [24]. Such approach is necessary to assess trade-offs of acoustic niche partitioning for long-distance communication signals in the species of the temperate austral forest.

In the current study we conducted recordings of the dissimilar calls produced by individual males of *E*. *calcaratus* and *E*. *emiliopugini* within a shared native environment to assess the relative effectiveness of their sound communication systems by means of precise measurements of attenuation and degradation patterns of signals composing a partitioned acoustic niche.

## Materials and Methods

### Ethical statements

The procedures used in this study comply with the laws of animal welfare in Chile (Bioethics Animal Committee, Faculty of Medicine, University of Chile, Protocol CBA# 061 FMUCH). The National Forestry Corporation (CONAF) authorized our field work in the Vicente Pérez Rosales National Park (Permit 09/2012).

### Field call recordings

#### Study site

The study was conducted in 2011 and 2012, during the months of September and October (*E*. *calcaratus*) and November and December (*E*. *emiliopugini*), at the locality of La Picada (41° 06' S, 72° 30' W, altitude 820 m above sea level), within the Vicente Pérez Rosales National Park in southern Chile. The study site was a bog of volcanic substrate, where males of *E*. *calcaratus* and *E*. *emiliopugini* call from inside small burrows along the borders of small streams or pools among vegetation composed mainly of mosses (*Rhacomytrium)*, grasses, (*Scyrpus* and *Myrteola*) and ferns (*Blechnum*). Previous studies on the vocal behavior of both species have been carried out at this site [27–35].

#### Call recording procedures

Field measurements were carried out placing four miniature omnidirectional microphones (Sennheiser MKE 2, K6 power supply) at different distances from the opening of a burrow occupied by a calling male at ground level. The microphones were placed on the substrate surface and connected to a Tascam DR 680 digital recorder. Two arrays of microphones were used to record vocalizations from each frog: in microphone array 1, the microphones were placed at 0.25, 0.5, 1 and 4 m, and in microphone array 2, the microphones were at 0.25, 2, 4 and 8 m. Calls of 15 *E*. *calcaratus* and of 11 *E*. *emiliopugini* were recorded using this procedure during 2011 and calls of other 6 *E*. *emiliopugini* were recorded during 2012, totaling 17 subjects for this species.

At the end of a recording session, a 1-kHz tone from a sound calibrator (Brüel & Kjaer 4230) was recorded with the microphones at the same gain level used for call recordings to provide a signal of a known SPL value (93.8 dB SPL RMS) to calculate the SPLs of the calls recorded in the four channels.

Air and substrate temperatures were measured after recording the calls of each animal with a thermocouple thermometer (Digi-Sense 8528–20) to the nearest 0.1°C. Air humidity was measured with a precision psychrometer (Extech Instruments RH 390) to the nearest 0.1%.

### Signal analysis

#### Amplitude analysis

Ten calls recorded for a given subject at four microphone positions devoid of interfering environmental sounds were chosen for analysis. To achieve this, the initial and final times of each call were measured using Raven Pro 1.4 (Cornell Lab of Ornithology, Bioacoustics Research Program). These selections were used for signal analysis with a custom-automated analysis implemented with R (version 3.0.2; [36]) and the sound analysis package Seewave (version 1.7.2; [37]).

Calls of *E*. *calcaratus* recorded at 8 m from the emitters were embedded in noise and therefore, corrections were applied to subtract the background noise following Bradbury and Vehrencamp1998, pages 34–35, [38]. These modifications were done measuring the RMS amplitude of intervals contiguous to the calls analyzed containing just background noise.

Transmission losses for propagating calls were calculated as follows: values predicted by spherical spreading were computed with the equation: spherical transmission loss (dB) = 20 log10 (far distance (m)/0.25 (m)), and this value was subtracted from the actual transmission loss, i. e. the decrease in average SPL from 0.25 m from the loudspeaker to the corresponding farther distances. Positive and negative values indicated that the sound attenuated at higher and lower rates, respectively, relative to the SPLs predicted by spherical transmission loss for each distance (positive or negative excess attenuation).

#### Spectral analysis

**Fig 1:**
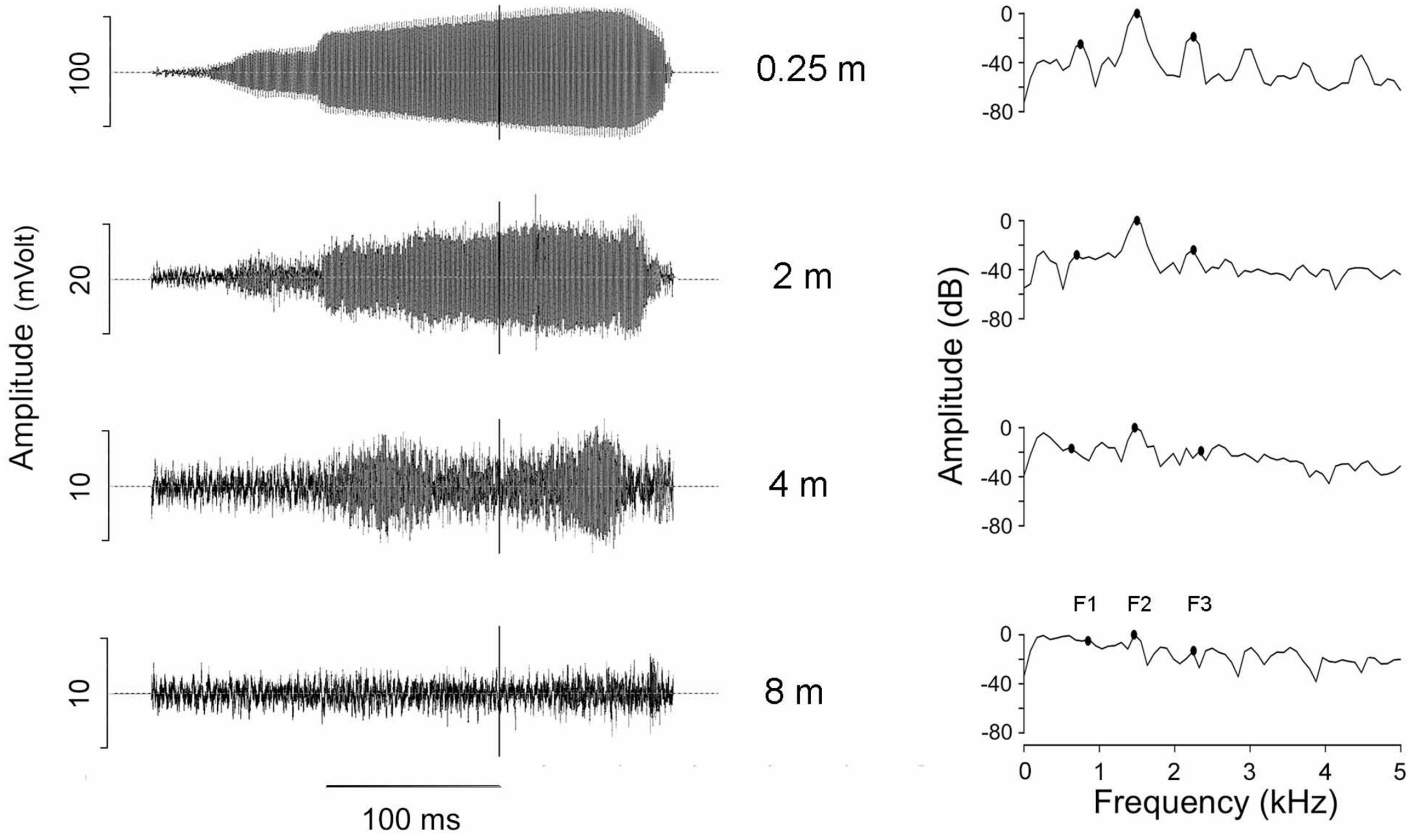
Oscillograms (left column) and power spectra (right column, 512 points, 86.13 Hz bandwidth) of a call of an *Eupsophus calcaratus* male recorded at different distances from its burrow opening (air temperature: 2.1°C, substrate temperature: 6.6°C). This recording was obtained using microphone array 2 (see Material and Methods). The vertical lines on the oscillograms indicate the time at which the power spectra were obtained. The black dots on the power spectra indicate the position of three first harmonics: F1, F2 and F3.

For *E*. *calcaratus*, power spectra were obtained at the time equaling two thirds of the duration of a call, which corresponded typically to the time of maximum amplitude of the signal (see Fig 1). A fast Fourier transform with a window length of 512 points (frequency resolution: 86.13 Hz, temporal resolution: 11.61 ms) was used. From the power spectra obtained, the frequency and the relative amplitude of the first (F1), second (F2) and third (F3) harmonics were measured. The amplitude values were used to calculate the amplitude ratios F2/F1 and F2/F3. In addition, the power spectra were used to calculate the spectral cross-correlations between the calls at 0.25 m and the farther distances (0.5, 1.0, 2.0, 4.0 and 8.0 m).

**Fig 2:**
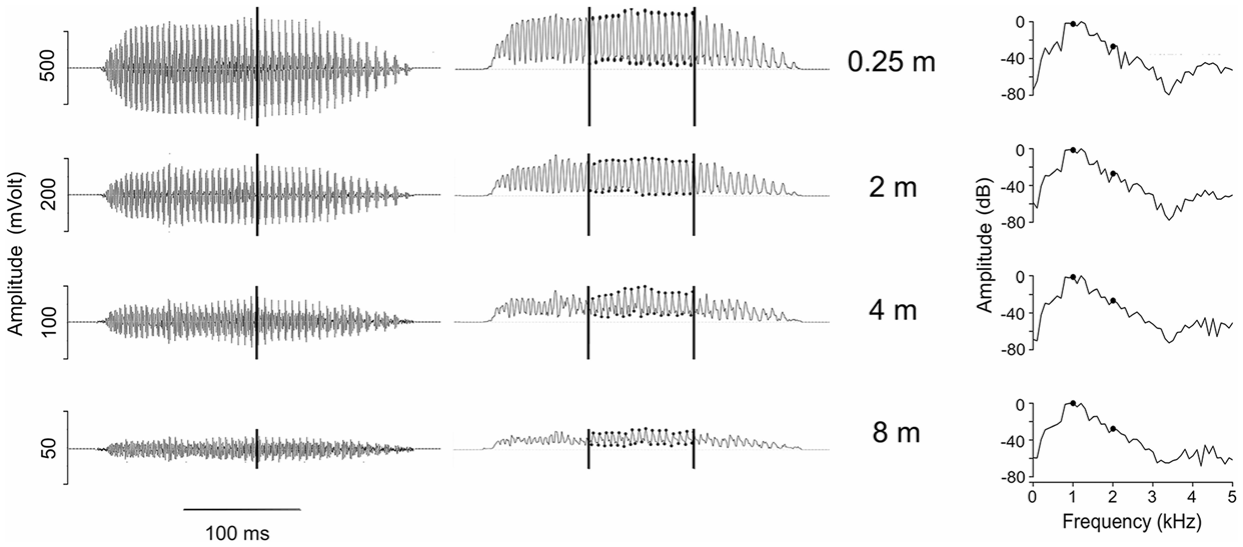
Oscillograms (left column), amplitude envelope (middle column) and power spectra (right column, 440 points, 100.23 Hz bandwidth) of a call of an *Eupsophus emiliopugini* male recorded at different distances from its burrow opening (air temperature: 8.7°C, substrate temperature: 9.1°C). This recording was obtained using microphone array 2 (see Material and Methods). The vertical lines on the oscillograms indicate the time at which the power spectra were obtained. The black dots on the amplitude envelope indicate the interval at the central segment of the call at which these values were measured (see text). The black dots on the power spectra indicate the frequencies of 1 and 2 kHz for which the amplitudes were measured.

For *E*. *emiliopugini*, power spectra were obtained at the mid time of a call duration, which corresponded typically to the time of maximum amplitude of the signal (see Fig 2). A fast Fourier transform with a window length of 440 points (frequency resolution: 100.23 Hz, temporal resolution: 9.98 ms) was used. This frequency resolution allowed us to obtain the relative amplitude of spectra at 1.0, and 2.0 kHz to calculate the amplitude ratios between the spectral components at these frequencies.

#### Amplitude modulation analysis

Because the call of *E*. *emiliopugini* has a pulsed structure, measurements of amplitude modulation depth of this signal were done obtaining the envelope of calls using a smoothing of 30 points and a 0% overlap between windows applied at the mid third of the calls analyzed, as shown in Fig 2. The maximum and minimum envelope values of the amplitude modulation were obtained to calculate the modulation depth percentage.

For the interspecific comparison of call spectra the same settings used for *E*. *calcaratus* were employed (window length: 512 points, frequency resolution: 86.13 Hz, temporal resolution: 11.61 ms).

### Statistical analysis

To determine the effects of distance on each call variable of each species we fitted linear mixed effects models by maximum likelihood using the R (version 3.0.2; [36]) library “lme4” (version 1.0–4; [39]). This analysis was used because each individual was recorded simultaneously along different distances, and therefore data dependence must be taken into account for statistical analysis [40]. Individual intercepts were included as a random effect, however, change in trend across distance was not included as a random effect because of model convergence issues. The distance was included as a fixed effect and its significance was evaluated with a likelihood ratio test (LRT). If the inclusion of the distance was supported, the model was refitted using restricted maximum likelihood. *A priori* contrasts were performed to determine distances at which calls differed from calls at 0.25 m. Confidence intervals with Bonferroni correction were obtained from these contrasts to determine if a zero value occurred within its range, which indicated a lack of significant differences between paired distances [40]. This analysis was done separately for each microphone array, and variables related to amplitude (i. e. SPL and excess attenuation) were fitted in linear scale. Nevertheless, log- or square root- transformations were performed in order to improve normality.

To determine the trends of change of acoustic variables for each species as calls propagated we tested for different trends of variation: linear, exponential, maximum or minimum values at about the middle of the recording transect, and no change along distance. For this analysis the distance was considered as a continuous predictor variable and the individual as a random effect. These models were fitted using the R library “nlme” (version 3.1.111; [41]). This library was used because it contains a more complete documentation on non-linear model fitting than “lme4” [40, 42]. We first plotted the raw data to obtain a general idea of the trend of each acoustic variable. Then we tested the presumed model against a simpler model through LRTs. This analysis was done using all the recordings of microphone array 1 (i.e. 25, 50, 100 and 400 cm) and the complementary distances of microphone array 2 (i.e. 200 and 800 cm). In this analysis we included random effects for each model parameter, unless model convergence issues occurred.

For comparison between species linear mixed-effects models were fitted using the R (version 3.0.2; [36]) library “lme4” (version 1.0–4; [39]). As for the previous analysis, individual intercepts were included as random effects. Species, distance, and their interaction were included as fixed effects and their significance was evaluated through LRTs.

## Results

### General features

The advertisement calls of 16 males of *E*. *calcaratus* recorded at 0.25 m consisted of a single note having an average duration of 285 ms (range 247–356 ms), and a harmonic structure with the three first components at averages of 791, 1492 and 2198 Hz (ranges 698–869, 1335–1628 and 1964–2386 Hz, respectively). The second harmonic was the spectral component having the largest average amplitude at 0.25 m, the first and third harmonic were about 35 and 10 dB below this component, respectively. The amplitude of the advertisement calls of *E*. *calcaratus* calls decreased from about 75 dB SPL RMS at 0.25 m to about 50 and 40 dB SPL RMS at 4 and 8 m, respectively. At this farther location the amplitude of the signals fell close to the background noise level (average signal-to-noise ratio: 2.49 dB, range: 0.3–8.7 dB) and therefore corrections were applied as described in Methods, so that the call amplitudes were reduced by 2.65 dB on average (range: 0.6–4.0 dB). No corrections were applied for recordings at other distances, because the signal-to-noise ratio was above 10 dB at these microphone locations, ratios at which modifications are not justified [43]. A call of an individual male recorded at different distances is shown in Fig 1.

The advertisement calls of 17 males of *E*. *emiliopugini* recorded at 0.25 m consisted of pulsed notes having an average duration of 245 ms (range 127–293 ms), and spectra with side-bands having a dominant frequency at an average of 1138 Hz (range 563–1302 Hz, respectively). The amplitude of the advertisement calls of *E*. *emiliopugini* decreased from about 85 dB SPL RMS at 0.25 m to about 58 and 50 dB SPL RMS at 4 and 8 m, respectively. At this farther location the amplitude of the signals was well above the background noise level, so corrections were not applied as for *E*. *calcaratus*. A call of an individual male recorded at different distances is shown in Fig 2.

### E. calcaratus

**Fig 3:**
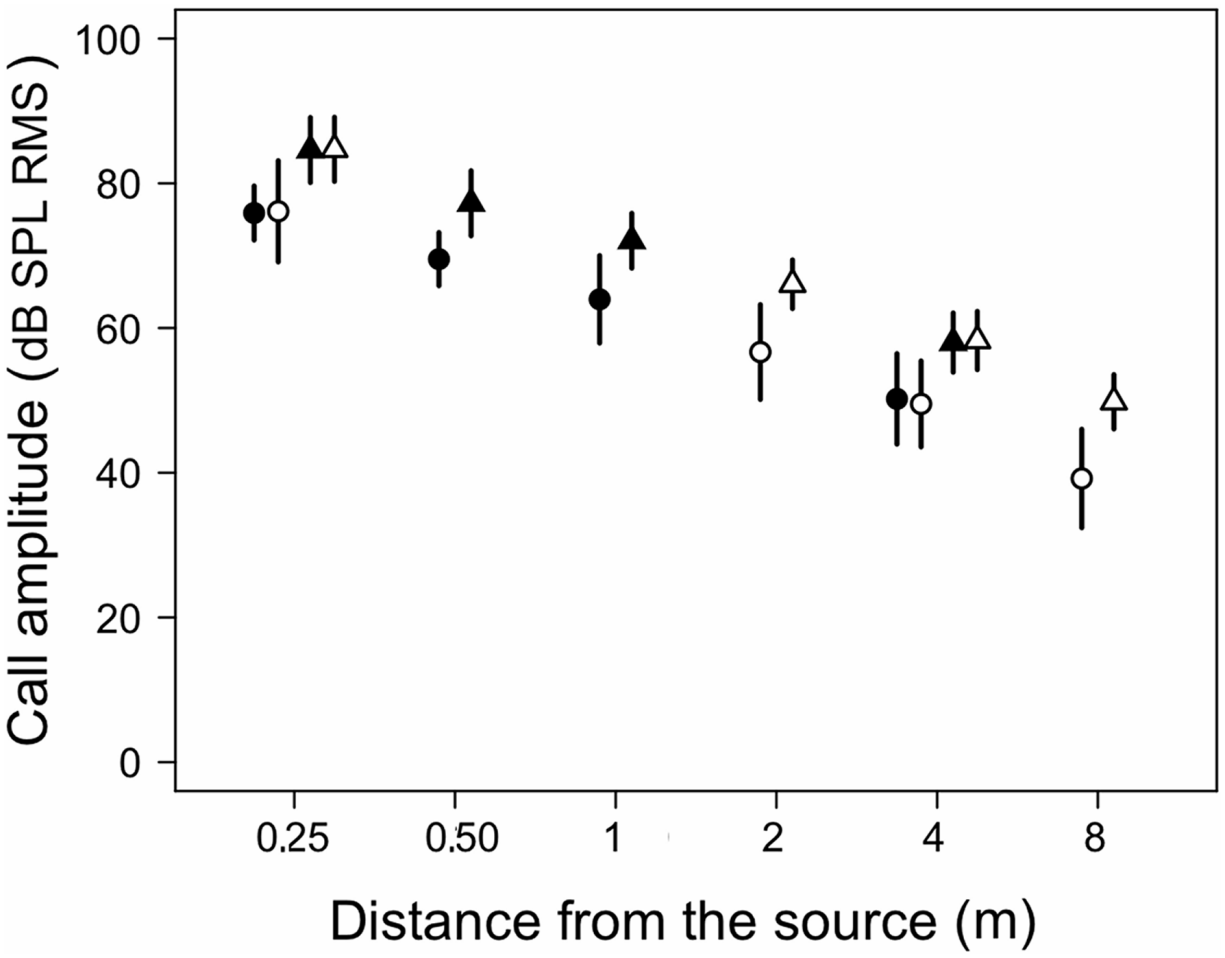
Sound pressure levels of advertisement calls recorded at different distances from calling males. Circles and triangles indicate averages of measurements for calls of males of *Eupsophus calcaratus* (N = 15) and *E*. *emiliopugini* (N = 17), respectively. Filled and open symbols correspond to microphones array 1 and 2, respectively (see text). Bars indicate standard deviations.

**Fig 4:**
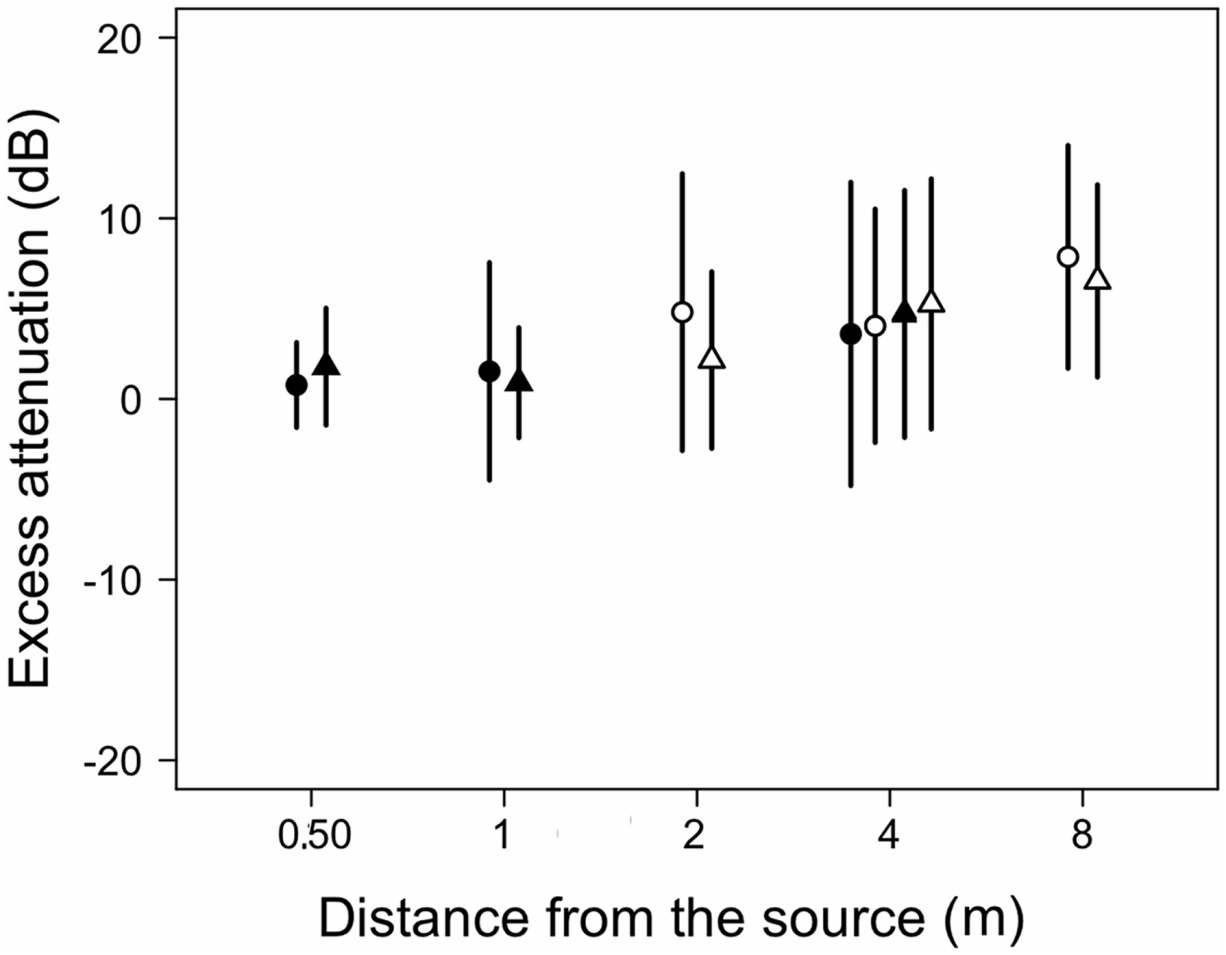
Excess attenuations relative to SPLs measured at 0.25 m from emitters for advertisement calls recorded at different distances from calling males. Symbols as in Fig 3.

Analyses of propagation patterns of the advertisement calls of *E*. *calcaratus* showed a significant effect of distance on SPL for microphone arrays 1 and 2 (χ2 = 130.2, df = 3, p < 0.0001 and χ2 = 138.3, df = 3, p < 0.0001, respectively). Significant differences occurred between SPLs measured at 0.25 m and measurements at all the farther distances for microphone arrays 1 and 2 (Fig 3, S1 Table). The SPLs of *E*. *calcaratus* calls decreased exponentially as a function of distance (χ2 = 57.7, df = 4, p < 0.0001; dB SPL = 35.42+40.25*e^(-0.00336*dist)^. Excess attenuation of calls at 0.5 m were on average about 1 dB and about 4 and 8 dB at 4 and 8 m, respectively. These values did not differ significantly between distances for microphone array 1 (χ2 = 3.4, df = 2, p = 0.1811) but differed significantly for microphone array 2 (χ2 = 9.7, df = 2, p = 0.0080). Excess attenuation measured at 8 m was significantly higher than at 2 m for microphone array 2 (Fig 4, S1 Table). Excess attenuation increased linearly with distance (χ2 = 23.3, df = 3, p < 0.0001; EA dB = 0.0263+0.0076*dist).

**Fig 5:**
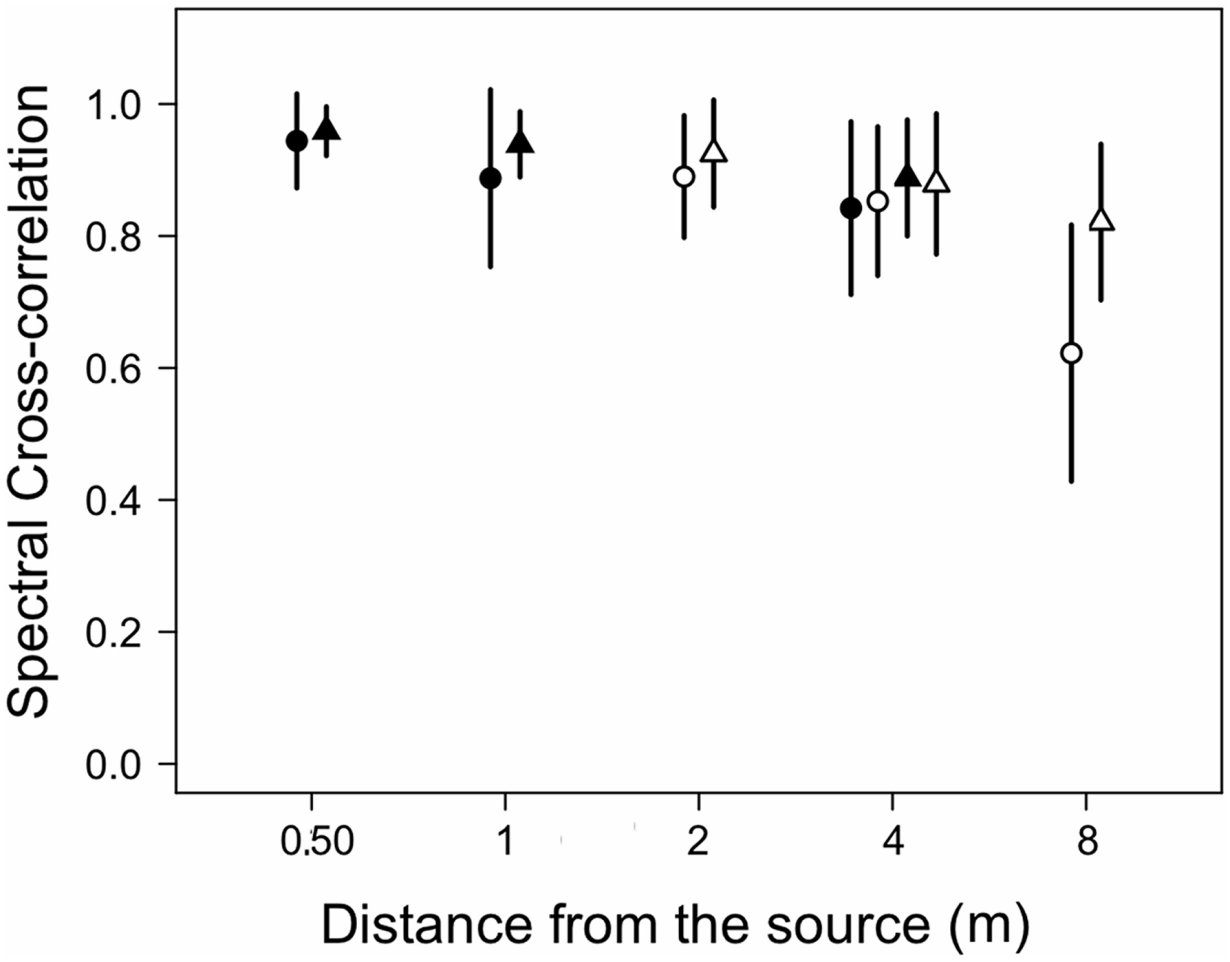
Spectral cross correlations for advertisement calls recorded at different distances from calling males. Symbols as in Fig 3.

Spectral changes measured as cross-correlation values between call spectra at 0.25 m and at farther distances were affected by distance in both microphone arrays (microphone array 1: χ2 = 14.6, df = 2, p = 0.0007; microphone array 2: χ2 = 32.2, df = 2, p < 0.0001), showing values averaging about 0.95 at 0.5 m and about 0.85 and 0.60 at 4 and 8 m, respectively (Fig 5). For microphone array 1, the reference cross-correlation value obtained at 4 m was significantly lower than the value measured at 0.5 m. For microphone array 2, the reference cross-correlation value obtained at 8 m was significantly lower than the value measured at 2 m (S1 Table). The cross-correlation value decreased linearly as a function of distance (χ2 = 59.6, df = 3, p < 0.0001), CC = 0.95940–0.00040*dist.

**Fig 6:**
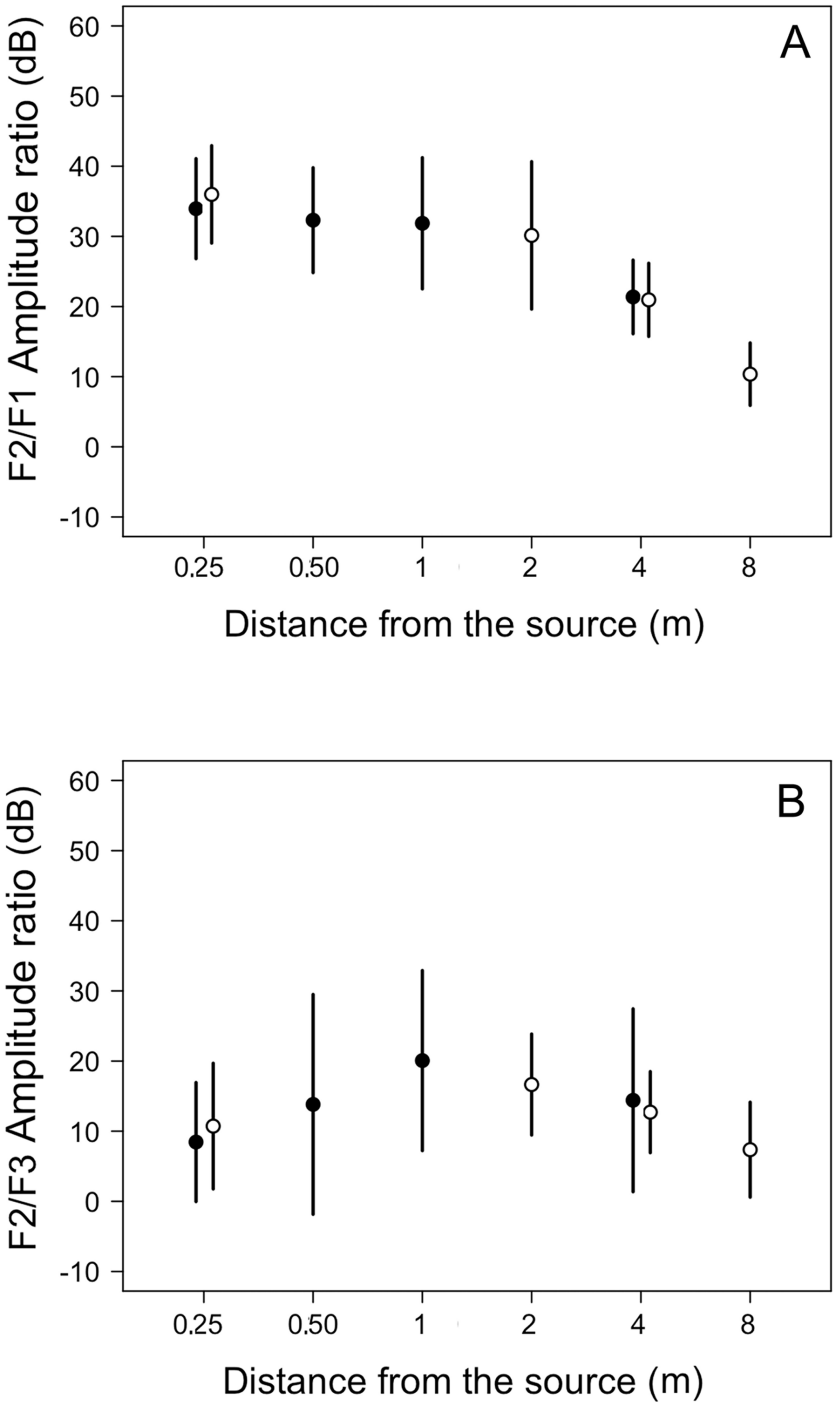
Amplitude ratios between spectral components of calls of 15 males of *E*. *calcaratus*. **A: Amplitude ratio between the second (F2) and first (F1) harmonic.** B: Amplitude ratio between the second (F2) and third (F3) harmonic.

The spectral structure of the calls measured as harmonic amplitude ratios was affected by distance as well: the F2/F1 amplitude ratio decreased from about 35 dB at 0.25 m to about 20 and 10 dB at 4 and 8 m, respectively (Fig 6a). The F2/F1-amplitude ratio differed among distances for microphone arrays 1 and 2 (χ2 = 35.2, df = 3, p < 0.0001 and χ2 = 51.9, df = 23 p < 0.0001, respectively). The F2/F1 ratio was significantly lower at 4 m relative to 0.25 m for microphone array 1 and lower at 4 and 8 m relative to 0.25 m for microphone array 2 (Fig 6a, S1 Table). The F2/F1 amplitude ratio decreased linearly as a function of distance (χ2 = 95.8, df = 3, p < 0.0001; F2/F1 dB = 30.50–0.02688*dist).

The F2/F3 amplitude ratio was on average about 10 dB at 0.25 m, reached about 20 dB at 1 m and decreased to about 14 dB and 7 dB at 4 and 8 m, respectively (Fig 6b). This ratio differed among distances for microphone arrays 1 and 2 (χ2 = 20.0, df = 3, p = 0.0002 and χ2 = 18.8, df = 3, p = 0.0003, respectively). The F2/F3 amplitude ratio was significantly larger at 1 and 4 m relative to 0.25 m for microphone array 1 and significantly larger at 2 and 4 m relative to 0.25 m for microphone array 2 (Fig 6b, S1 Table). The F2/F3 amplitude ratio showed a trend having a maximum at an intermediate distance along the recording transect (χ2 = 12.7, df = 1, p = 0.0004; F2/F3 dB = 5.05+0.03493*dist-0.00004*dist2).

### E. emiliopugini

Analyses of propagation patterns of the advertisement calls of *E*. *emiliopugini* indicate a significant effect of distance on SPL for microphone arrays 1 and 2 (χ2 = 173.9, df = 3, p < 0.0001 and χ2 = 184.3, df = 3, p < 0.0001, respectively). Values at 0.25 m were significantly larger than those at all the farther distances for microphone arrays 1 and 2 (Fig 3, S2 Table). The SPL decreased exponentially as a function of distance (χ2 = 118.5, df = 4, p < 0.0001; dB SPL = 47.39+37.60*e^(-0.00397*dist)^).

Excess attenuation of calls at 0.5 m was on average about 2 dB and increased to about 5 and 7 dB at 4 and 8 m, respectively (Fig 4). Significant differences occurred among distances for microphone arrays 1 and 2 (χ2 = 9.8, df = 2, p = 0.0073 and χ2 = 21.3, df = 2, p < 0.0001, respectively). In microphone array 1 significant differences did not occur in the *a priori* contrasts. In microphone array 2, excess attenuation was significantly higher at 4 and 8 m relative to 2 m (S2 Table). The EA dB increased linearly with distance (χ2 = 39.2, df = 1, p < 0.0001; EA dB = 0.34902+0.00628*dist).

Spectral changes measured as cross-correlation values between call spectra at 0.25 m and at farther distances were also affected by distance showing on average values of about 0.95 at 0.5 m and decreasing to about 0.88 and 0.82 at 4 and 8 m, respectively (Fig 5). Significant differences occurred for microphone arrays 1 and 2 (χ2 = 14.2, df = 2, p = 0.0008 and χ2 = 21.9, df = 2, p < 0.0001, respectively. For microphone array 1, the reference cross-correlation value obtained at 4 m was significantly lower than the value measured at 0.5 m. For microphone array 2, the reference cross-correlation value obtained at 4 and 8 m was significantly lower than value measured at 2 m. The cross-correlation value decreased linearly as a function of distance (χ2 = 56.4, df = 3, p < 0.0001; CC = 0.96128–0.00018*dist).

**Fig 7:**
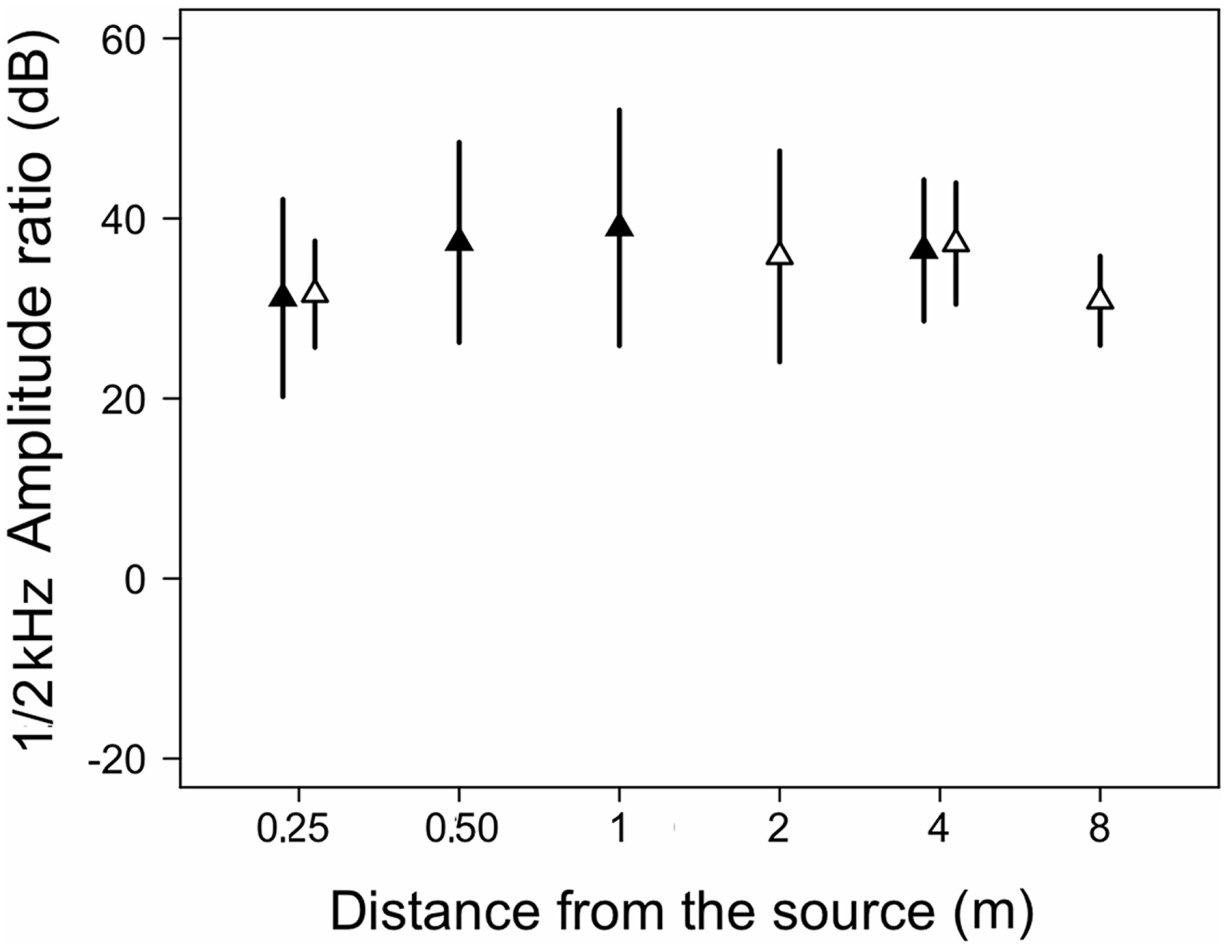
Amplitude ratios between spectra of calls of 17 males of *E*. *emilipugini* at 1 and 2 kHz.

The spectral structure of the calls measured as amplitude ratios between two frequencies was affected by distance as well: the ratio of spectral amplitudes between frequencies 1–2 kHz was on average about 31 dB at 0.25 m, increased to about 37 dB at distances 1–4 m and decreased to about 30 dB at 8 m (Fig 7). The amplitude ratio 1–2 kHz differed among distances for microphone arrays 1 and 2 (χ2 = 12.1, df = 3, p = 0.007 and χ2 = 9.4, df = 3 p = 0.02, respectively). The amplitude ratio was significantly higher at 1 and 4 m relative to 0.25 m for microphone array 1. In microphone array 2 significant differences did not occur in the *a priori* contrasts. The 1–2 kHz dB ratio had a trend with a maximum at an intermediate distance (χ2 = 11.9, df = 4, p = 0.0183; 1–2 kHz dB = 29.07+0.02329*dist-0.00003*dist2).

**Fig 8:**
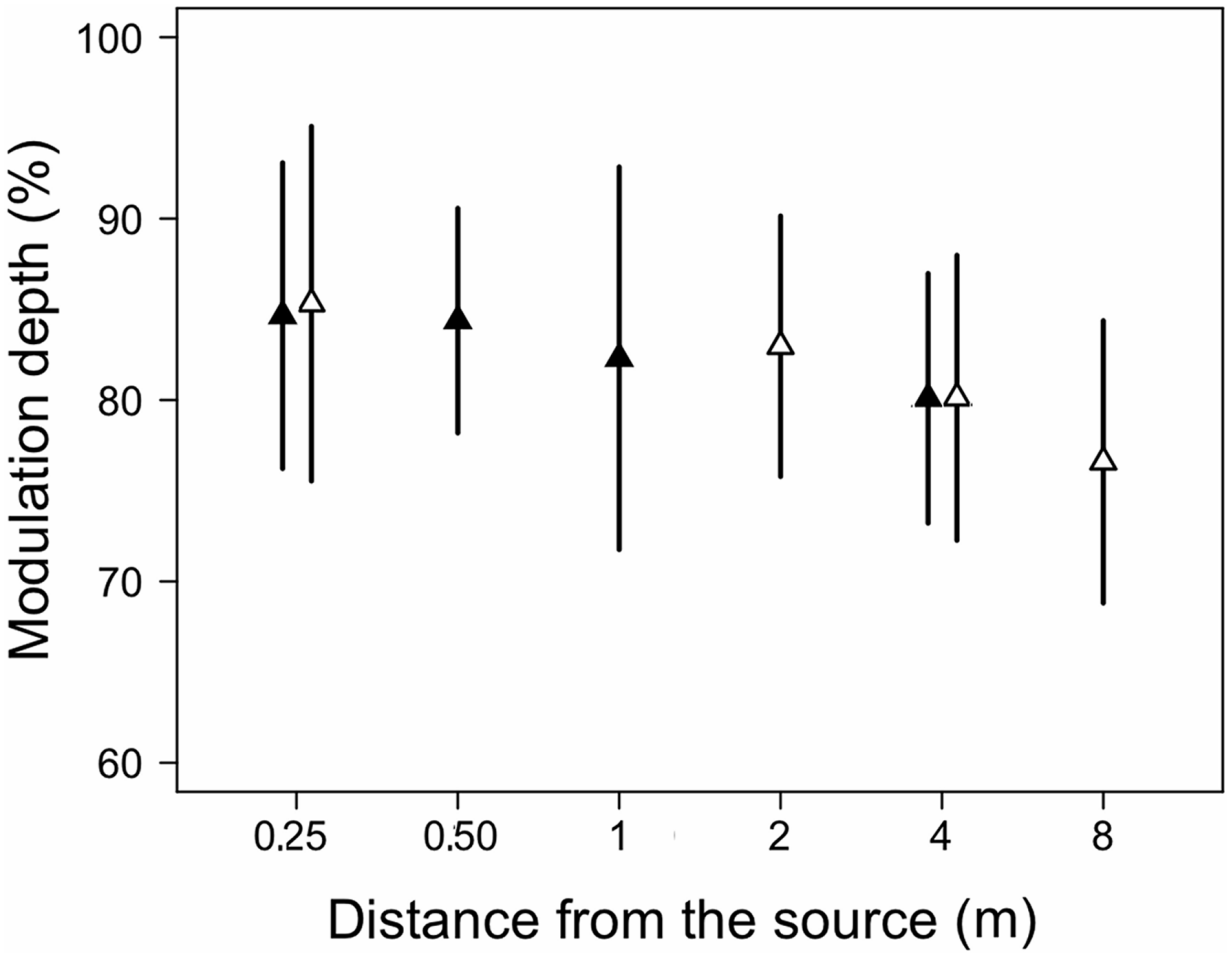
Amplitude modulation of calls of 17 males of *E*. *emilipugini* recorded at different distances from the burrow openings.

The modulation depth of the pulses of the calls was on average about 85% at 0.25 m and decreased to about 80 and 76% at 4 and 8 m, respectively (Fig 8). Distance had a significant effect on this variable for microphone arrays 1 and 2 (χ2 = 11.4, df = 3, p = 0.0097 and χ2 = 33.1, df = 3 p < 0.0001, respectively). *A priori* contrasts showed that modulation depth was lower at 4 m relative to 0.25 m for microphone array 1 and lower at 4 and 8 m relative to 0.25 m for microphone array 2. The modulation depth decreased linearly as a function of distance (χ2 = 39.6, df = 3, p < 0.0001; MD = 84.46–0.01004*dist).

### Inter-specific comparative analysis

#### Sound pressure level

The sound pressure level was significantly different between species (χ2 = 25.6, df = 1, p < 0.0001) and among distances (χ2 = 481.2, df = 5, p < 0.0001). The interaction between species and distance was not significant (χ2 = 5.0, df = 5, p = 0.4188)

#### Excess attenuation

The excess attenuation did not differ between species (χ2 = <0.1, df = 1, p = 0.9439) but distance had a significant effect (χ2 = 57.4, df = 4, p < 0.0001). No interaction between both factors occurred (χ2 = 4.7, df = 4, p = 0.3207).

#### Spectral cross-correlation

The spectral cross-correlation was significantly affected by species (χ2 = 4.7, df = 1, p = 0.0305) and distance (χ2 = 95.5, df = 4, p < 0.0001). The interaction between species and distance was statistically significant as well (χ2 = 14.8, df = 4, p = 0.0052).

## Discussion

The results of this study show differences in the propagation of signals of the two species analyzed that are in general agreement with two recent studies [27, 28]: the calls of *E calcaratus* have low SPLs relative to *E*. *emiliopugini* and the calls of the first species are not detectable within the background noise at 8 m from the sound source, whereas the calls of the second species are clearly detectable at this distance.

The non-linear decrease of SPLs with distance in both species is in agreement with spherical propagation of sound in the atmosphere, which accounts for a 6-dB SPL decrease per two-fold increases in distance from the sound source. The additional transmission losses measured as excess attenuation are similar for calls of both species and likely originate in scattering and ground effects acting on signals of relatively low frequency contents transmitted at ground level over short distances [44–48]. The linear mode of increase in excess attenuation with distance in both species indicates that losses dependent on scattering and ground effects over the distances considered do not affect signal propagation to the extent reported for high-frequency signals of insects, for which excess attenuations proportional to two-fold increases in distance have been reported [49].

These results of transmission losses show general similarity with measurements on propagation of vocalizations of Iberian toads *Alytes* [24], which yielded average excess attenuations of about 5 dB at 4 and 8 m from the sound source. However the values obtained both in the current as in the study of Iberian anurans are variable among individuals. In both studies, excess attenuations are predominantly positive, but negative values also occur. This variability is likely dependent on the topography and vegetation coverage of the transects along which measurements were carried out for the different individuals.

Spectral cross-correlation differs between species, distance and the interaction between these factors, and decreases linearly with distance. Studies in insects (e. g. [50]) and anurans (e.g. [10]) have revealed that spectral cross-correlation is a useful measure to account for signal degradation depending on spectral contents, as higher frequency components degrade at higher rates. In *E*. *calcaratus*, our detailed analysis indicates that the F2/F1 amplitude ratio decreases linearly, meaning that the amplitudes of these harmonics tend to equalize at longer distances. Contrastingly, the F2/F3 amplitude ratio increases at intermediate distances but then decreases at farther distances along the recording transect. This relationship shows that at intermediate distances higher frequencies are attenuated preferentially but all frequencies in the 1400–2100 Hz range are similarly affected at the farther distances recorded. In *E*. *emiliopugini* the 1/2 kHz amplitude ratio shows a trend similar to the one observed for the F2/F3 ratio of *E*. *calcaratus*. It is likely that these complex trends combining linear and non-linear dynamics in spectral degradation restrict the receivers’ assessment of distance from the sound source based on spectral cues.

The decrease in modulation depth affecting the call of *E*. *emiliopugini* could provide a cue for estimating the emitter’s distance in this species. The linear mode of variation bears general similarity to variation in amplitude modulation of calls of the North American *Hyla chrysoscelis*, for which both linear and exponential decrements have been reported to occur at distances of 8 m from the sound source [51].

The SPLs of the calls of *E*. *calcaratus* at distant positions from the sound source are higher relative to those measured previously for this species [27]. At 4 m the SPLs in the current study were at about 50 dB SPL whereas in the former study were about 40 dB SPL. Measurements in the current study are likely more accurate than those of the previous study due to the larger sample size and because they were carried out simultaneously with recordings at closer distances. Also two sets of measurements at this distance were obtained for each individual. As measured in the former study [27], the SPL corresponding to the auditory thresholds for conspecific advertisement calls are about 58 dB SPL, so calls would not be detectable at this distance. SPLs of the calls of this frog reach about 57 at 2 m, which supports the conclusion of that study limiting the communication range of this frog at distances shorter than 2 m. The restricted active space of *E*. *calcaratus* relative to *E*. *emiliopugini* results from the lower amplitude of the call of the first species and not from different attenuation rates of the signals transmitted in the breeding areas, since the excess attenuations are similar between both species.

Overall this study shows that two anurans breeding and communicating in the very same microenvironment with signals having contrasting structure—i.e. tonal harmonic in *E*. *calcaratus* and pulsed with spectral sidebands in *E*. *emiliopugini–*, also differ in the mode in which the spectral structure of these calls, as measured by spectral cross-correlation is affected by the physical properties of the breeding area. Previous studies have shown that these species also differ in the strategies for confronting abiotic noise [32, 34], as the vocal activity of *E*. *calcaratus* is enhanced in the presence of noise, whereas the vocal output of *E*. *emiliopugini* remains unaltered or decreases when confronted with the same interferences. The microenvironment in which the two species compared breed and call is fairly similar: occasionally individuals of both taxa occupy the same burrows in successive breeding seasons [28]. Also the breeding seasons of the two taxa overlap partially and no detectable changes in vegetation structure occur throughout the reproductive periods of both species in these evergreen landscapes.

The various characteristics in which the acoustic communication systems between these two anurans differ are likely related to their evolutionary history, as phylogenetic relationships classify *E*. *calcaratus* and *E*. *emiliopugini* apart in the Roseus and Vertebralis group of *Eupsophus*, respectively [52]. This condition is supported by the occurrence of a consistent phylogenetic signal in anuran advertisement calls [15]. The call divergence between these syntopic frogs contributes support for a lack of relationship of signal structure and communication strategies with environmental conditions in anurans, complementing evidence drawn from comparisons of the transmission of signals of various anuran taxa native from different geographical distributions and environments (reviewed in [18, 19]).

The divergence in the communication system between *E*. *calcaratus* and *E*. *emiliopugini* contributes to acoustic niche partitioning, a condition that has been reported formerly for other species assemblages in the temperate austral forest [53, 54]. Our results indicate that acoustic partitioning has dissimilar consequences for the structural changes of propagating signals between the two species. Studies to evaluate the relevance of the spectral and temporal properties affected by linear changes with distance for recognition by calling males are currently underway.

Further measurements of signal propagation considering additional *Eupsophus* species and localities are needed for an accurate assessment of the relative importance of phylogenetic constraints and selective agents.

## Supporting Information

S1 Table A priori contrasts of variables to estimate differences between distances for propagating calls of *E*. *calcaratus*. Confidence intervals were obtained with Bonferroni corrections.
(DOCX)

S2 Table A priori contrasts of variables to estimate differences between distances for propagating calls of *E*. *emiliopugini*. Confidence intervals were obtained with Bonferroni corrections.
(DOCX)
