# Supplementary material for: Contrasting Propagation of Natural Calls of Two Anuran Species from the South American Temperate Forest
Source: PLoS One. 2015 Jul 31;10(7):e0134498. doi: 10.1371/journal.pone.0134498 (PMC4521761; doi:10.1371/journal.pone.0134498)
Supplement: S1 Table — Confidence intervals were obtained with Bonferroni corrections. (DOCX) [file pone.0134498.s001.docx]

**S1 Table.** A priori contrasts of variables to estimate differences between distances for propagating calls of *E. calcaratus.* Confidence intervals were obtained with Bonferroni corrections.

| Variable | Contrasts between distances (m) | Estimated difference | CI low | CI up |
| --- | --- | --- | --- | --- |
| SPL-1 (dB SPL) | 0.5-0.25 | -6.509 | -9.015 | -4.004 |
|  | 1.0-0.25 | -12.421 | -14.926 | -9.915 |
|  | 4.0-0.25 | -26.201 | -28.706 | -23.695 |
|  |  |  |  |  |
| SPL-2 (dB SPL) | 2.0-0.25 | -20.422 | -23.897 | -16.946 |
|  | 4.0-0.25 | -26.599 | -30.075 | -23.123 |
|  | 8.0-0.25 | -36.721 | -40.197 | -33.245 |
|  |  |  |  |  |
| EA-1 (dB) | 1.0-0.5 | -0.089 | -2.555 | 2.377 |
|  | 4.0-0.5 | 1.691 | -0.775 | 4.158 |
|  |  |  |  |  |
| EA-2 (dB) | 4.0-2.0 | 0.178 | -2.905 | 3.260 |
|  | 8.0-2.0 | 3.938 | 0.856 | 7.021 |
|  |  |  |  |  |
| F2/F1-1 (dB) | 0.5-0.25 | -1.106 | -5.378 | 3.166 |
|  | 1.0-0.25 | -1.669 | -5.941 | 2.602 |
|  | 4.0-0.25 | -11.019 | -15.290 | -6.747 |
|  |  |  |  |  |
| F2/F1-2 (dB) | 2.0-0.25 | -5.335 | -10.845 | 0.175 |
|  | 4.0-0.25 | -12.002 | -17.512 | -6.493 |
|  | 8.0-0.25 | -20.437 | -25.947 | -14.928 |
|  |  |  |  |  |
| F2/F3-1 (dB) | 0.5-0.25 | 2.656 | -1.910 | 7.222 |
|  | 1.0-0.25 | 8.612 | 4.045 | 13.178 |
|  | 4.0-0.25 | 5.947 | 1.381 | 10.513 |
|  |  |  |  |  |
| F2/F3-2 (dB) | 2.0-0.25 | 7.748 | 2.985 | 12.511 |
|  | 4.0-0.25 | 4.768 | 0.005 | 9.531 |
|  | 8.0-0.25 | 0.124 | -4.639 | 4.887 |
|  |  |  |  |  |
| CC-1 | 1.0-0.5 | -0.123 | -0.255 | 0.011 |
|  | 4.0-0.5 | -0.245 | -0.376 | -0.113 |
|  |  |  |  |  |
| CC-2 | 4.0-2.0 | -0.088 | -0.234 | 0.060 |
|  | 8.0-2.0 | -0.432 | -0.561 | -0.294 |

Abbreviations:

CI: Confidence intervals

SPL: Call sound pressure level

EA: Call excess attenuation

F2/F1: Amplitude ratio between call harmonics 2 and 1

F2/F3: Amplitude ratio between callharmonics 2 and 3

CC: Call spectral cross correlation

-1: Microphone array 1 (25. 50. 100 and 400 cm)

-2: Microphone array 2 (25. 200. 400 and 800 cm)
