# Supplementary material for: Contrasting Propagation of Natural Calls of Two Anuran Species from the South American Temperate Forest
Source: PLoS One. 2015 Jul 31;10(7):e0134498. doi: 10.1371/journal.pone.0134498 (PMC4521761; doi:10.1371/journal.pone.0134498)
Supplement: S2 Table — Confidence intervals were obtained with Bonferroni corrections. (DOCX) [file pone.0134498.s002.docx]

**S2 Table**. A priori contrasts of variables to estimate differences between distances for propagating calls of *E. emiliopugini.* Confidence intervals were obtained with Bonferroni corrections.

| Variable | Contrasts between distances (m) | Estimated difference | CI low | CI up |
| --- | --- | --- | --- | --- |
| SPL-1 (dB SPL) | 0.5-0.25 | -7.310 | -9.037 | -5.583 |
|  | 1.0-0.25 | -12.505 | -14.232 | -10.777 |
|  | 4.0-0.25 | -26.627 | -28.385 | -24.861 |
|  |  |  |  |  |
| SPL-2 (dB SPL) | 2.0-0.25 | -19.079 | -21.308 | -16.850 |
|  | 4.0-0.25 | -27.058 | -29.287 | -24.829 |
|  | 8.0-0.25 | -35.324 | -37.554 | -33.095 |
|  |  |  |  |  |
| EA-1 (dB) | 1.0-0.5 | -0.806 | -2.275 | 0.664 |
|  | 4.0-0.5 | 1.364 | -0.140 | 2.861 |
|  |  |  |  |  |
| EA-2 (dB) | 4.0-2.0 | 2.668 | 0.836 | 4.501 |
|  | 8.0-2.0 | 4.246 | 2.413 | 6.078 |
|  |  |  |  |  |
| MD-1 (%) | 0.5-0.25 | -0.275 | -3.803 | 3.252 |
|  | 1.0-0.25 | -1.348 | -4.934 | 2.265 |
|  | 4.0-0.25 | -4.558 | -8.085 | -1.030 |
|  |  |  |  |  |
| MD-2 (%) | 2.0-0.25 | -2.346 | -5.672 | 0.979 |
|  | 4.0-0.25 | -5.188 | -8.514 | -1.863 |
|  | 8.0-0.25 | -8.721 | -12.046 | -5.395 |
|  |  |  |  |  |
| R ½-1 (dB) | 0.5-0.25 | 4.069 | -0.610 | 8.747 |
|  | 1.0-0.25 | 6.568 | 1.889 | 11.246 |
|  | 4.0-0.25 | 5.413 | 0.735 | 10.091 |
|  |  |  |  |  |
| R ½-2 (dB) | 2.0-0.25 | 4.409 | -0.968 | 9.787 |
|  | 4.0-0.25 | 5.343 | -0.035 | 10.720 |
|  | 8.0-0.25 | -0.078 | -5.455 | 5.300 |
|  |  |  |  |  |
| CC-1 | 1.0-0.5 | -0.061 | -0.147 | 0.025 |
|  | 4.0-0.5 | -0.158 | -0.244 | -0.070 |
|  |  |  |  |  |
| CC-2 | 4.0-2.0 | -0.108 | -0.199 | -0.016 |
|  | 8.0-2.0 | -0.218 | -0.307 | -0.127 |

Abbreviations:

CI: Confidence intervals

SPL: Call sound pressure level

EA: Call excess attenuation

MD: Amplitude modulation depth

R 1/2: Amplitude ratio between 1 and 2 kHz

CC: Call spectral cross correlation

-1: Microphone array 1 (25, 50, 100 and 400 cm)

-2: Microphone array 2 (25, 200, 400 and 800 cm)
